# Effects of transcranial magnetic stimulation on sleep structure and quality in children with autism

**DOI:** 10.3389/fpsyt.2024.1413961

**Published:** 2024-06-28

**Authors:** Juan Yan, Yan Zhang, Junjie Wang, Guidong Zhu, Kaijie Fang

**Affiliations:** ^1^ Quality Control Office, Hangzhou Seventh People’s Hospital, Hangzhou, Zhejiang, China; ^2^ Administration Office, Lishui Second People’s Hospital, Lishui, Zhejiang, China

**Keywords:** autism spectrum disorder, transcranial magnetic stimulation, sleep quality, dorsolateral prefrontal cortex, children

## Abstract

**Introduction:**

Sleep disorders are common in children with autism spectrum disorder (ASD). Transcranial magnetic stimulation (TMS) can influence the excitability of neuronal cells in stimulated areas, leading to improvements in sleep and other autistic symptoms. However, studies on clinical mechanisms of TMS in treating sleep disorders associated with ASD are limited. Therefore, we aimed to explore the effects of TMS on sleep structure and quality in children with ASD.

**Methods:**

Between January 2020 and December 2021, recruitment was advertised through child and adolescent outpatient clinics and online platforms by the Hangzhou Seventh People’s Hospital and Lishui Second People’s Hospital. Sixty children with ASD who met the diagnostic criteria of the Diagnostic and Statistical Manual of Mental Disorders, Fifth Edition, were selected and randomly divided into the active TMS and sham TMS treatment groups. Thirty healthy children of the same age were recruited as controls. The active TMS group received bilateral low-frequency (0.5 Hz) TMS targeting the dorsolateral prefrontal cortex on both sides in children with ASD, whereas the sham TMS group received sham stimulation with the same stimulation time and location as the experimental group. Both groups were treated for 6 weeks, and the participants were assessed using the Sleep Disturbance Scale for Children (SDSC) before treatment, at 3 weeks, and at 6 weeks of intervention. Independent sample *t-*tests and difference *t*-tests were used for statistical analysis of the data.

**Results:**

No significant differences were observed in general demographic variables, such as age and sex, between the ASD and control groups (*P*>0.05). Independent sample *t*-test analysis showed that the total SDSC score, difficulty falling asleep, sleep maintenance, awakening disorders, sleep-wake transition disorders, excessive daytime sleepiness, and nocturnal hyperhidrosis scores were significantly higher in the ASD group than in the control group (*P*<0.05). Before treatment, no significant differences were observed in the factor or total SDSC scores between the sham TMS group and the active TMS group (*P*>0.05). After 15 and 30 treatment sessions, the total SDSC score, difficulty falling asleep, sleep maintenance, sleep-wake transition disorders, and excessive daytime sleepiness scores were significantly higher in the sham TMS group than in the active TMS group (*P*<0.05). The difference t-test analysis showed that after 30 treatment sessions, the reduction rates of the total SDSC score, difficulty falling asleep, sleep maintenance, awakening disorders, sleep-wake transition disorders, excessive daytime sleepiness, and nocturnal hyperhidrosis dimensions were significantly higher in the active TMS group than in the sham TMS group (*P*<0.05).

**Conclusion:**

Low-frequency TMS targeting the dorsolateral prefrontal cortex in children with ASD can effectively improve their sleep status, and significant improvement can be achieved after 6 weeks (30 sessions) of treatment.

## 1 Introduction

Autism spectrum disorder (ASD) is a complex neurodevelopmental condition characterized primarily by persistent deficits in social communication and interactions across multiple contexts, along with restricted and repetitive patterns of behavior, interests, and activities (1, 2). According to the 2015 “Report on the Development of Autism Education and Rehabilitation Industry in China,” it is estimated that there may be over 10 million individuals with ASD in China, with > 2 million children aged 0–14 years affected (3). Furthermore, data from the latest National Survey of Children’s Health in the United States in 2016 (4) revealed a prevalence rate of 2.79% among children aged 3–17 years, whereas the prevalence rate of ASD in Chinese children was approximately 0.265% (5).

In addition to core symptoms, children with ASD often exhibit one or more comorbid conditions, including intellectual disability, sleep disorders, epilepsy, and gastrointestinal dysfunction (6, 7). Sleep disorders are particularly common in children with ASD and manifest as prolonged sleep latency, increased sleep arousal, reduced slow-wave sleep, decreased total sleep time, and reduced sleep efficiency. In turn, insufficient sleep exacerbates the core symptoms of ASD (such as repetitive and stereotypical behaviors and social impairments) (8, 9) and other maladaptive behaviors (such as self-injury, aggression, hyperactivity, disobedience, irritability, and emotional issues) (10–15).

Currently, the primary treatment methods for sleep disorders in individuals with ASD include pharmacological therapy, behavioral interventions, and alternative therapies. Although pharmacological therapy and behavioral interventions can improve sleep disorders to varying degrees in children with ASD, there are concerns regarding the potential side effects associated with drug treatment. Additionally, the heterogeneity among children with ASD and the limitations in implementing behavioral interventions, specifically for this population, make it challenging to select the most appropriate intervention measures. Behavioral interventions may not be suitable for younger children. Therefore, there is a pressing need to identify safe, rapid, and tolerable treatment methods to improve sleep quality in this population.

Transcranial magnetic stimulation (TMS) is a neuromodulation technique that uses Faraday’s law of electromagnetic induction to generate time-varying pulsed magnetic fields that penetrate the skull and stimulate the brain. This stimulation alters the membrane potential of neuronal cells in the cerebral cortex, inducing excitatory (at frequencies > 1 Hz, known as high frequency) or inhibitory (at frequencies < 1 Hz, known as low-frequency) effects (16). Research (17–21) has demonstrated that TMS targeting the dorsolateral prefrontal cortex can influence the excitability of neuronal cells in stimulated areas, leading to improvements in anxiety, depression, sleep, repetitive behaviors, and social impairments. However, studies on the clinical mechanisms of TMS in the treatment of sleep disorders associated with ASD are limited.

Therefore, this study aimed to investigate the effects of low-frequency TMS on sleep structure and quality in children with ASD aged 8–15 years who experienced sleep disorders. Specifically, TMS will be used to stimulate the dorsolateral prefrontal cortex bilaterally with the aim of providing evidence for further improving the sleep status of children with ASD.

## 2 Material and methods

### 2.1 Participants

Between January 2020 and December 2021, recruitment was advertised through child and adolescent outpatient clinics and online platforms at the Seventh People’s Hospital of Hangzhou and the Second People’s Hospital of Lishui. This study recruited 60 children aged 8–15 years with ASD and sleep disorders. Additionally, 30 healthy children in the same age range were recruited as the controls.

The inclusion criteria were as follows: (1) age between 8 and 15 years; (2) right-handedness; (3) no speech, hearing, or vision impairments; (4) no history of severe physical illness or nervous system infection; (5) no use of central nervous system drugs for at least one week before assessment; and (6) fulfillment of the diagnostic criteria for autism in the Diagnostic and Statistical Manual of Mental Disorders, Fifth Edition.

Exclusion criteria included: (1) inability to complete the intervention tests; (2) history of epilepsy; (3) history of brain injury, brain tumor, encephalitis, or brain metabolic disorders; and (4) presence of metal objects in their bodies. This study was approved by the ethics management committee of the hospital, and patients or their guardians voluntarily agreed to participate. The legal guardians of all the patients included in the study signed informed consent forms.

### 2.2 Assessment scales

The Sleep Disturbance Scale for Children (SDSC), which has good reliability and validity (22), was used to assess sleep quality in children aged 6–15 years. Either the child or the main caregiver responded to the questionnaire, and the adults filled out the forms. The SDSC comprises 26 items across six dimensions: difficulties in initiating and maintaining sleep, sleep-disordered breathing, disorders of arousal, sleep-wake transition disorders, daytime excessive sleepiness, and sleep hyperhidrosis (Night Sweats). Higher scores indicate more severe sleep disturbance. Bruni et al. (23) set a total score of 39 as the cutoff value, which corresponds to the upper quartile of the control group. The sensitivity and specificity of this cutoff were 0.89 and 0.74, respectively. Therefore, an SDSC total score > 39 was considered indicative of sleep disturbance.

### 2.3 Quality control

Before the trial, the researchers underwent training in questionnaire administration and TMS techniques (Transcranial Magnetic Stimulator, S-50, Shenzhen Yingchi Technology Co., Ltd.) to ensure uniform and standardized treatment procedures and minimize measurement bias. During stimulation, the participants wore electrode caps to confirm the stimulation location. Data entry was performed using the double-entry method.

### 2.4 Statistical analysis

Statistical analysis was conducted using SPSS 25.0. Continuous variables are expressed as mean ± standard deviation (SD). Independent sample *t*-tests were used to compare the groups. Differences were considered statistically significant at *P* < 0.05.

### 2.5 Research methods

#### 2.5.1 TMS intervention protocol

Participants with ASD were randomly divided into two groups: the active TMS group, and the sham TMS group, with 30 participants in each group. No statistically significant differences were observed between the groups in terms of various indicators or general characteristics, including age, sex, disease duration, education level, and personality traits. We used the Five Centimeter Rule to identify participants’ DLPFC (24). To ensure data rigor and single-factor variable control, excluding placebo effects, the following intervention methods were adopted:

Normal age-matched control group: No treatment was administered.

The active TMS group: Received real TMS stimulation (100% MT, 0.5 Hz, 300 pulses per side) once daily for 6 weeks (five sessions per week) (25).

The sham TMS group: Received sham TMS stimulation (5% MT, 0.5 Hz, 300 pulses per side) once daily for 6 weeks (five sessions per week).

#### 2.5.2 Outcome assessment

Assessments were conducted using the SDSC, sleep structure analysis, cortical neural excitability measures, and neural network indicators after 15 and 30 treatment sessions.

## 3 Result

### 3.1 General demographic characteristics

Sixty children with ASD were surveyed in this study, with a mean age of 11.20 ± 2.313 years. Among them, 31 were males and 29 were females. Additionally, 30 typically developing children were included, averaging 11.90 ± 2.139 years old, with 12 males and 18 females. Ninety questionnaires were distributed to both the ASD and control groups, with all 90 questionnaires successfully recovered, resulting in 100% questionnaire efficiency. The results indicated no statistically significant differences in age or sex between the ASD and control groups (P>0.05) (**Table 1**).

**Table 1:** Comparison of baseline data between the ASD and normal control groups ( $\bar{x}$ ± *s*, n(%)).

Demographic Variables		Normal Control Group (n=30)	TMS sham-treated Group (n=30)	TMS-treated Group (n=30)	F
Gender	Male	12	12	19	
	Female	18	18	11	
Age		11.90 ± 2.139	11.27 ± 2.392	11.13 ± 2.270	0.977

### 3.2 Comparison of sleep disorders between the ASD group and the normal control group

Independent sample t-tests were conducted to analyze sleep disorders in children with ASD and typically developing children. The results revealed that the ASD group had significantly higher total SDSC scores, difficulties in falling asleep and maintaining sleep, arousal disorders, sleep-wake transition disorders, excessive somnolence, and nocturnal hyperhidrosis compared to the normal group (P<0.05), as shown in **Table 2**.

**Table 2:** Comparison of SDSC scores between the ASD and normal control groups ( $\bar{x}$ ± *s*).

Dimensions	ASD Group (60)	Normal Control Group (n=30) S	t-Value	p-Value
Difficulties in Initiating and Maintaining Sleep	26.27 ± 3.579	10.57 ± 2.329	-25.001	0.000
Sleep-Related Respiratory Disorders	3.35 ± 0.633	3.13 ± 0.346	-2.098	0.039
Awakening Disorders	3.90 ± 0.933	3.07 ± 0.254	-6.455	0.000
Sleep-Wake Transition Disorders	13.87 ± 2.855	8.27 ± 0.980	-13.667	0.000
Excessive Daytime Sleepiness	11.15 ± 2.349	6.83 ± 1.341	-11.074	0.000
Nocturnal Hyperhidrosis	4.27 ± 1.177	3.40 ± 1.567	-2.676	0.010
SDSC Score	62.80 ± 8.931	35.27 ± 2.227	-22.521	0.000

### 3.3 Comparison of sleep disorders between the sham TMS group and the active TMS group before treatment

All participants completed 30 sessions of treatment without any dropouts due to adverse effects. Independent sample t-tests were used to compare sleep disorders between the sham TMS group and the active TMS group before treatment. The results showed that there were no significant differences in SDSC total score, difficulties in falling asleep and maintaining sleep, arousal disorders, sleep-wake transition disorders, excessive somnolence, and nocturnal hyperhidrosis between the two groups (P>0.05), as detailed in **Table 3**.

**Table 3:** Comparison of SDSC scores between the TMS sham-treated and TMS-treated groups after treatment ( $\bar{x}$ ± *s*).

Dimensions	Sessions of treatment	TMS sham-treated Group (n=30)	TMS-treated Group (n=30)	t-value	p-value
Difficulties in Initiating and Maintaining Sleep	0	26.33 ± 3.252	26.20 ± 3.934	0.143	0.887
	15	25.37 ± 3.615	21.53 ± 4.006	3.891	0.000
	30	24.63 ± 3.068	18.23 ± 3.287	7.796	0.000
Sleep-Related Respiratory Disorders	0	3.37 ± 0.615	3.33 ± 0.661	0.202	0.840
	15	3.23 ± 0.504	3.27 ± 0.521	-0.252	0.802
	30	3.30 ± 0.466	3.27 ± 0.521	0.261	0.795
Awakening Disorders	0	3.93 ± 1.015	3.87 ± 0.860	0.274	0.785
	15	3.77 ± 0.679	3.63 ± 0.850	0.671	0.505
	30	3.63 ± 0.615	3.50±+0.630	0.830	0.410
Sleep-Wake Transition Disorders	0	13.80 ± 2.833	13.93 ± 2.924	-0.179	0.858
	15	13.20 ± 2.683	11.83 ± 2.214	2.152	0.036
	30	13.03 ± 2.327	11.37 ± 2.236	2.829	0.006
Excessive Daytime Sleepiness	0	11.27 ± 2.333	11.03 ± 2.399	0.382	0.704
	15	10.93 ± 2.083	9.77 ± 1.832	2.303	0.025
	30	10.87 ± 2.300	9.20 ± 1.690	3.198	0.002
Nocturnal Hyperhidrosis	0	4.20 ± 1.270	4.33 ± 1.093	-0.436	0.665
	15	4.00 ± 1.145	3.93 ± 0.828	0.258	0.797
	30	3.90 ± 0.995	3.83 ± 0.699	0.300	0.765
SDSC Score	0	62.90 ± 8.794	62.70 ± 9.214	0.086	0.932
	15	60.50 ± 7.877	53.97 ± 7.384	3.314	0.002
	30	59.37 ± 6.744	49.40 ± 5.721	6.173	0.000

### 3.4 Comparison of sleep disorders between the sham TMS group and the active TMS group after treatment

According to the F and P analysis before and after treatment, the active TMS group showed significant therapeutic effects compared to the sham TMS group, with the effects of Maintaining Sleep, Sleep-Wake Transition Disorders, and Excessive Daytime Sleepiness being particularly significant (P<0.05), as detailed in **Tables 4**, **5**.

**Table 4:** The F and P analysis before and after 15 treatment sessions.

		Average	F	P
Maintaining Sleep	1	26.33 ± 3.252	1.980	0.144
	2	25.37 ± 3.615		
	3	24.63 ± 3.068		
Sleep-Related Respiratory Disorders	1	3.37 ± 0.615	0.471	0.626
	2	3.23 ± 0.504		
	3	3.30 ± 0.466		
Awakening Disorders	1	3.93 ± 1.015	1.088	0.341
	2	3.77 ± 0.679		
	3	3.63 ± 0.615		
Sleep-Wake Transition Disorders	1	13.80 ± 2.833	0.709	0.495
	2	13.20 ± 2.683		
	3	13.03 ± 2.327		
Excessive Daytime Sleepiness	1	11.27 ± 2.333	0.274	0.761
	2	10.93 ± 2.083		
	3	10.87 ± 2.300		
Nocturnal Hyperhidrosis	1	4.20 ± 1.270	0.537	0.587
	2	4.00 ± 1.145		
	3	3.90 ± 0.995		
SDSC Score	1	62.90 ± 8.794	1.585	0.211
	2	60.50 ± 7.877		
	3	59.37 ± 6.744		

The F and P analysis before and after 15 treatment sessions.

**Table 5:** The F and P analysis before and after 30 treatment sessions.

		Average	F	P
Maintaining Sleep	1	26.20 ± 3.934	34.065	0.000
	2	21.53 ± 4.006		
	3	18.23 ± 3.287		
Sleep-Related Respiratory Disorders	1	3.33 ± 0.661	0.136	0.873
	2	3.27 ± 0.521		
	3	3.27 ± 0.521		
Awakening Disorders	1	3.87 ± 0.860	1.667	0.195
	2	3.63 ± 0.850		
	3	3.50 ± 0.630		
Sleep-Wake Transition Disorders	1	13.93 ± 2.924	9.9119	0.000
	2	11.83 ± 2.214		
	3	11.37 ± 2.236		
Excessive Daytime Sleepiness	1	11.03 ± 2.399	6.625	0.002
	2	9.77 ± 1.832		
	3	9.20 ± 1.690		
Nocturnal Hyperhidrosis	1	4.33 ± 1.093	2.659	0.076
	2	3.93 ± 0.828		
	3	3.83 ± 0.699		
SDSC Score	1	62.70 ± 9.214	23.875	0.000
	2	53.97 ± 7.384		
	3	49.40 ± 5.721		

The F and P analysis before and after 30 treatment sessions.

Independent sample t-tests were conducted to analyze sleep disorders in the sham TMS group and the active TMS group after 15 and 30 treatment sessions. The findings revealed that the sham TMS group had higher scores in SDSC total score, difficulties in falling asleep and maintaining sleep, sleep-wake transition disorders, and excessive somnolence compared to the active TMS group, with statistically significant differences (P<0.05), as summarized in **Table 3**.

### 3.5 Comparison of sleep disorders between the ASD and control groups

Difference t-tests were used to assess sleep disorders in the sham and active TMS groups after 30 treatment sessions. The results indicated that the active TMS group had significantly higher reduction rates in SDSC total score, difficulties in falling asleep and maintaining sleep, arousal disorders, sleep-wake transition disorders, excessive somnolence, and nocturnal hyperhidrosis than the sham TMS group (*P*<0.05) (**Table 6**)

**Table 6:** Comparison of SDSC score reductions between the TMS sham-treated and TMS-treated groups after 30 sessions of treatment ( $\bar{x}$ ± *s*).

Dimensions	TMS sham-treated Group (n=30)	TMS-treated Group (n=30)	t-value	p-value
Difficulties in Initiating and Maintaining Sleep	-1.70 ± 2.200	-7.97 ± 2.748	15.88	0
Sleep-Related Respiratory Disorders	-0.07 ± 0.785	-0.07 ± 0.365	1	0.321
Awakening Disorders	-0.30 ± 0.877	-0.37 ± 0.718	2.796	0.007
Sleep-Wake Transition Disorders	-0.77 ± 1.073	-2.57 ± 1.888	7.446	0
Excessive Daytime Sleepiness	-0.40 ± 0.968	-1.83 ± 1.341	7.487	0
Nocturnal Hyperhidrosis	-0.30 ± 0.877	0.50 ± 0.938	2.921	0.005
SDSC Score	-3.53 ± 3.277	-13.30 ± 5.011	14.536	0

## 4 Discussion

Adequate sleep is a crucial safeguard for children’s brain development, information processing, cognitive function, memory, and learning capabilities (26). Numerous domestic and international studies have demonstrated that the prevalence of sleep disorders in children with ASD is higher than that in typically developing children, ranging from approximately 44–86% (6, 27–29), while the prevalence in typically developing children is approximately 25–40% (30–32). The prevalence of sleep disorders in children and adolescents with ASD is approximately two to three times higher than that in their typically developing peers. A sleep survey conducted by Li Shiyun et al. in China among 84 children with ASD aged 3–12 years and 91 typically developing children revealed that children with ASD exhibited more difficulties in initiating and maintaining sleep, reduced total sleep time, poorer sleep quality, and night awakenings than typically developing children, which is consistent with the findings of this study.

TMS is a novel neuromodulatory therapy that regulates neural excitability, accelerates synaptogenesis, and modulates neurotransmitter transmission and expression by applying a pulsed magnetic field to the cerebral cortex. In this study, TMS was administered to children with ASD, and the results showed that the total efficacy rate was significantly higher in the active TMS group than in the sham TMS group (P < 0.05). We found that the sleep quality was better after 30 TMS treatment sessions than after 15 sessions, which is consistent with previous studies that reported that 20 TMS treatment sessions showed significant improvement (33). Studies have demonstrated that 6 consecutive weeks (five sessions per week) of low-frequency bilateral TMS stimulation of the dorsolateral prefrontal cortex resulted in significant improvements in sleep structure and functional connectivity strength in different brain regions.

Currently, most studies on insomnia interventions employ low-frequency (1 Hz) TMS to stimulate the right dorsolateral prefrontal cortex (16). In this study, low-frequency (0.5 Hz) TMS was used to stimulate the dorsolateral prefrontal cortex bilaterally in children with ASD. The results revealed improvements in sleep disorders (SDSC factor scores, total scores, and reduction rates) in the active TMS group, whereas no statistically significant changes were observed in the control group that received sham stimulation (*P*<0.05). This suggests that TMS has a specific effect on patients with ASD and sleep disorders. The therapeutic mechanism may be related to improving the imbalance between excitation and inhibition commonly observed in patients with ASD and enhancing the excitation-inhibition ratio (34–36). Foreign studies have suggested that low-frequency stimulation of the right dorsolateral prefrontal cortex can improve sleep through mechanisms such as increased serum levels of brain-derived neurotrophic factor and gamma-aminobutyric acid and reduced motor-evoked potentials.

This study had some limitations, such as its relatively small sample size and focus only on the dorsolateral prefrontal cortex. Future studies should address these shortcomings. The age range of the participants was set at 8–15 years, without any subgroup analyses regarding the possible effects of puberty. These limitations will be addressed in future studies.

In summary, low-frequency bilateral TMS stimulation of the dorsolateral prefrontal cortex in children with ASD can effectively improve their sleep status, and significant improvements can be achieved after 6 weeks (30 sessions). Behavioral interventions are commonly used to treat sleep disorders in children with ASD. However, given the individual differences and potential side effects of pharmacological treatments, they are not recommended as first-line options unless the sleep disorder symptoms are severe. TMS is a novel, painless, side effect-free, safe, and effective alternative therapy that has been shown to have significant effects on sleep disorders. Therefore, TMS therapy may be considered for the treatment of sleep disorders in children with ASD.

## Data availability statement

The raw data supporting the conclusions of this article will be made available by the authors, without undue reservation.

## Ethics statement

The studies involving humans were approved by Ethics Committee of Lishui Second People’s Hospital. The studies were conducted in accordance with the local legislation and institutional requirements. Written informed consent for participation in this study was provided by the participants’ legal guardians/next of kin.

## Author contributions

JY: Conceptualization, Data curation, Writing – review & editing. YZ: Conceptualization, Funding acquisition, Writing – original draft. JW: Data curation, Methodology, Writing – original draft, Writing – review & editing. GZ: Data curation, Funding acquisition, Investigation, Writing – review & editing. KF: Writing – review & editing.
